# Irreversible inhibition of estrogen receptor α signaling and the emergence of hormonal resistance in MCF7 breast cancer cells induced by DNA damage agents

**DOI:** 10.3892/br.2024.1727

**Published:** 2024-01-19

**Authors:** Alexander M. Scherbakov, Danila V. Sorokin, Valeria E. Razuvaeva, Yuri Yu. Shchegolev, Olga E. Andreeva, Diana I. Salnikova, Timur I. Fetisov, Olga A. Vlasova, Kirill I. Kirsanov, Margarita V. Gudkova, Mikhail A. Krasil'nikov

**Affiliations:** 1 Department of Experimental Tumor Biology, Institute of Carcinogenesis, N.N. Blokhin National Medical Research Center of Oncology, The Ministry of Health of Russia, Moscow 115522, Russian Federation; 2 Molecular Genetics Laboratory, Institute of Clinical Medicine, National Research Lobachevsky State University of Nizhny Novgorod, Nizhny Novgorod 603022, Russian Federation; 3 Laboratory of Chemical Transformations of Antibiotics, Gause Institute of New Antibiotics, Moscow 119021, Russian Federation; 4 Department of Chemical Carcinogenesis, Institute of Carcinogenesis, N.N. Blokhin National Medical Research Center of Oncology, The Ministry of Health of Russia, Moscow 115522, Russian Federation; 5 Institute of Medicine, Patrice Lumumba Peoples' Friendship University of Russia, Moscow 117198, Russian Federation

**Keywords:** estrogen receptor α, breast cancer, DNA damage, tamoxifen, 5-fluorouracil, ultraviolet C irradiation, progression, resistance

## Abstract

Combining chemotherapy and hormone therapy is a prevalent approach in breast cancer treatment. While the cytotoxic impact of numerous chemotherapy drugs stems from DNA damage, the exact role of these DNA alterations in modulating estrogen receptor α (ERα) machinery remains elusive. The present study aimed to analyze the impact of DNA damage agents on ERα signaling in breast cancer cells and assess the signaling pathways mediating the influence of DNA damage drugs on the ERα machinery. Cell viability was assessed using the MTT method, while the expression of signaling proteins was analyzed by immunoblotting. ERα activity in the cells treated with various drugs (17β-estradiol, tamoxifen, 5-fluorouracil) was assessed through reporter gene assays. *In vitro* experiments were conducted on MCF7 breast cancer cells subjected to varying durations of 5-fluorouracil (5-FU) treatment. Two distinct cell responses to 5-FU were identified based on the duration of the treatment. A singular dose of 5-FU induces pronounced DNA fragmentation, temporally suppressing ERα signaling while concurrently activating AKT phosphorylation. This suppression reverses upon 5-FU withdrawal, restoring normalcy within ten days. However, chronic 5-FU treatment led to the emergence of 5-FU-resistant cells with irreversible alterations in ERα signaling, resulting in partial hormonal resistance. These changes mirror those observed in cells subjected to UV-induced DNA damage, underscoring the pivotal role of DNA damage in shaping estrogen signaling alterations in breast cancer cells. In summary, the results of the present study suggested that the administration of DNA damage agents to cancer cells can trigger irreversible suppression of estrogen signaling, fostering the development of partial hormonal resistance. This outcome may ultimately impede the efficacy of combined or subsequent chemo- and hormone therapy strategies.

## Introduction

The role of chemotherapy in the conservative treatment of malignant tumors is pivotal, representing a cornerstone in the therapeutic approach. The main objective in molecular oncology is the exploration of the mechanisms underlying chemotherapy-induced cellular changes and understanding the nature of cell death (1). In the past years, there has been an active exploration for agents and their synergistic combinations tailored to selectively target the pathways responsible for sustaining cancer resistance (2-5). The efficacy of combining different modalities of conservative therapy for breast cancer, especially the tandem use of chemotherapy and hormonal therapy, remains a largely unresolved question. While the impact of numerous chemotherapy drugs is linked to DNA damage, the precise role of these alterations in potentially influencing the estrogen receptor α (ERα) machinery and the hormonal response of tumors remains unclear. Current research in this domain heavily relies on the examination of clinical data, particularly the analysis of combined chemotherapy and hormone therapy effectiveness across diverse patient groups. However, findings in this area are often conflicting and contradictory. Specifically, evidence has revealed that incorporating tamoxifen into chemotherapy cycles enhances outcomes for ERα-positive breast cancer (6-9). Likewise, the combination of hormonal and chemotherapy treatments has been associated with improved survival among women aged over 60 years (10). Conversely, some studies have reported that additional hormonal therapy fails to yield a discernible impact on overall survival (8,11), while supplementary chemotherapy does not demonstrate enhanced outcomes when compared with hormonal therapy alone (12). Several studies have revealed changes in ERα status during neoadjuvant chemotherapy for breast tumors (13,14). Notably, a correlation has been established between neoadjuvant chemotherapy and increased expression of microRNA-18a, a member of the ERα suppressor family (15). The potential role of DNA damage in modulating ERα signaling was underscored in investigations exploring the effects of radiation on breast cancer. These studies revealed notable changes in hormonal signaling within irradiated cells (16,17).

In the present study, it was revealed for the first time that the treatment of MCF7 breast cancer cells with a single dose of 5-fluorouracil (5-FU) induces significant DNA fragmentation, which correlates with a transient suppression of estrogen signaling. Notably, continuous 5-FU treatment leads to the irreversible inhibition of ERα activity and the emergence of partial resistance to the antiestrogen tamoxifen. The pivotal role of DNA damage in altering estrogen signaling was further corroborated through parallel experiments involving ultraviolet-C (UVC)-irradiated cells. These irradiated cells exhibited a pronounced inhibition of estrogen machinery, mirroring the effects observed with 5-FU treatment. Chronic UVC irradiation, akin to prolonged 5-FU exposure, resulted in irreversible changes to estrogen receptor activity and a concomitant reduction in hormonal sensitivity. These findings strongly support the role of DNA damage in driving the progression of hormonal resistance.

## Materials and methods

### Cell cultures and reagents

Experiments were conducted on the MCF7 human breast cancer cell line (18) (cat. no. HTB-22^™^; American Type Culture Collection), authenticated by morphology and STR profiling through ‘Gordiz’ (http://gordiz.ru/, accessed on February 1, 2022). The cells were cultured at 37˚C with 5% CO_2_ in DMEM containing 4.5 g/l glucose (cat. no. СC420-02; PanEco), alanyl-glutamine (cat. no. Ф005; PanEco) and 7% fetal bovine serum (FBS) (cat. no. SV30160.03; HyClone; Cytiva). The response of the cells to tamoxifen (cat. no. 27190; Cayman Chemical Company) was assessed by treating them with tamoxifen for 3 days, followed by evaluating viability using the MTT assay [3-(4,5-dimethylthiazol-2-yl)-2,5-diphenyltetrazolium bromide] (cat. no. A2231; PanReac AppliChem) (19) modified as previously described (20). Dimethyl sulfoxide (DMSO) (cat. no. 191954; PanReac AppliChem) served as the solvent for the assay. Ultrapure water for the experiments was prepared using a Milli-Q water purification system (Merck KGaA).

### Treatment of MCF7 cells with 5-FU and the development of resistant clones

MCF7 breast cancer cells were seeded onto 24-well culture plates at a density of 40,000 cells per well. To assess cell sensitivity to 5-FU (cat. no. F6627; Sigma-Aldrich; Merck KGaA), the cells were exposed to 15 µM 5-FU for a 3-day period, followed by an analysis of the number of viable cells. To establish a 5-FU-resistant subline, MCF7 cells (at a density of 150,000 cells per well in 6-well plates) were cultured in DMEM medium with 7% FBS. The cells were exposed to increasing concentrations of 5-FU ranging from 5 to 30 µM over a span of 2 months, and this regimen was maintained for at least 1 month after withdrawal of 5-FU.

### UV irradiation and the selection of UV-resistant cells

Irradiation was conducted using 6W UV-lamp, emitting 254 nm light (model VL-6.LC; Vilber Lourmat). MCF7 cells were exposed to UVC irradiation (254 nm) at intensities of 50 J/m^2^. For the selection of UV-resistant cells, MCF7 cells were exposed to UVC once every three days for a duration of 4 weeks. Subsequently, cell growth was sustained for a minimum of 40 days following the conclusion of the last irradiation cycle.

### Colony-forming test

MCF7 cells were initially plated on 60-mm culture dishes at a density of 2 million cells per dish (Corning, Inc.). The following day, the DMEM culture medium was removed, and the seeded cells underwent UV irradiation (254 nm, 3 sec). After UV exposure, varying cell quantities were immediately seeded onto a 6-well culture plate (Corning, Inc.) in DMEM culture medium, aiming to establish 50-2,000 colonies per well. After a 14-day growth in a cell culture incubator, the colonies were fixed and stained using a solution of 20% methanol and 0.2% crystal violet at room temperature for 10 min. Any colony comprising >50 cells was identified and recorded as a viable surviving clone. Colonies were counted manually.

### Comet assay

The comet assay was conducted following established procedures outlined in a previous study (21). MCF7 cells were subjected to varying concentrations of 5-FU (15 and 30 µM) for a duration of 72 h or exposed to UVC irradiation, and subsequently, embedded in agarose on microscope slides. Following cell lysis and electrophoresis, the slides were stained with a DNA dye (SYBR Gold) for 5 min at room temperature. Observations were made using a Zeiss AxioVert 200 fluorescence microscope equipped with an EBQ isolated lamp at x10 magnification (Carl Zeiss AG). A minimum of 100 cells were captured for each sample and analyzed using CometScore 2.0 software (RexHoover) to quantify DNA damage.

### Micronucleus assay

To inhibit microfilament assembly and cytokinesis, cytochalasin B (cat. no. Х095; PanEco) was introduced into the medium at a final concentration of 6 µg/ml, 28 h prior to fixation across all experimental groups. Following cultivation, cells were collected, centrifuged at 1,200 x g for 10 min, and exposed to 0.075 M KCl (cat. no. 60129-100; PanEco) for 2 min. Subsequently, cells were fixed in ethanol-acetic acid (3:1), followed by another centrifugation at 1,000 x g at 4˚C for 7 min. The fixed cells were then transferred onto clean glass slides. All slides underwent staining with Giemsa solution (cat. no. 0080; PanEco) for 1 min at room temperature. Light microscopic analysis was performed on encrypted preparations, studying 2,000 binuclear cells from each group at a magnification of x400. The significance of differences in cytogenetic damage levels between control and treated cells was determined using Pearson's χ^2^ test. P<0.05 was considered to indicate a statistically significant difference.

### Transient transfection and the measurement of reporter gene activity

The transcriptional activity of ERα was assessed through reporter analysis, involving the transfection of ERE plasmids (luciferase-expressing reporter construct ERE-tk-LUC, which incorporates the estrogen response elements (EREs) from the vitellogenin A2 gene upstream of the thymidine kinase promoter) kindly provided by Professor George Reid from European Molecular Biology Laboratory (Heidelberg, Germany) (22,23). Transfection occurred under steroid-free conditions, utilizing DMEM without phenol red supplemented with 2% charcoal/dextran-treated fetal bovine serum (cat. no. SH30068.03; HyClone, Cytiva). This process was carried out for 6 h at 37˚C using Lipofectamine^®^ 2000 (Thermo Fisher Scientific, Inc.). For the transfection of a single well in Costar^®^ 24-well clear TC-treated plate (cat. no. 3524; Corning, Inc.), 0.8 µl of the transfection agent and 0.4 µg of plasmid DNA were employed. Co-transfection with a plasmid carrying the β-galactosidase gene served as a control to assess the efficiency and potential toxicity of the transfection process. 17β-Estradiol (E2) (cat. no. 3301; Sigma-Aldrich; Merck KGaA) in a concentration of 10 nM was used to treat cells during 24 h before determination of the luciferase and β-galactosidase activities. After 24 h post-transfection, cell lysis was carried out in 1x lysis buffer (cat. no. E1531; Promega Corporation), and luciferase activity was quantified using a Tecan Infinite M200 Pro luminometer (Tecan Group), following the manufacturer's protocol (Luciferase Assay System; cat. no. E1501; Promega Corporation) (24,25). β-Galactosidase activity was determined using ONPG (p-nitrophenyl β-D-galactopyranoside) (cat. no. 34055; Thermo Fisher Scientific, Inc.), the substrate for β-galactosidase. The cell lysates were combined with a phosphate buffer (pH 7.5, 0.1 M) containing ONPG (3.3 mM), MgCl_2_ (1 mM), and β-mercaptoethanol (53 mM). Absorbance at 405 nm was measured using the MultiScan FC reader (Thermo Fisher Scientific, Inc.) ERE reporter activity was calculated in arbitrary units as the luciferase/galactosidase activity ratio, following the method outlined in previous studies (24,25).

### Western blot analysis

To prepare samples for immunoblotting, cells were lysed in a buffer (150 µl) comprising Tris-HCl pH 7.4 (50 mM), Igepal CA-630 (1%), ethylenediamine tetraacetate (1 mM), dithiothreitol (1 mM), aprotinin, pepstatin and leupeptin (1 µg/ml), as well as sodium fluoride and sodium orthovanadate (1 mM). Protein content was determined using the Bradford method. Prior to centrifugation (10,000 x g, 10 min, 4˚C), the samples were incubated on ice for 20 min. Electrophoresis was performed on a 10% polyacrylamide gel, loaded with 60 µg of protein per lane, followed by protein transfer to a nitrocellulose membrane (Santa Cruz Biotechnology) and subsequent immunoblotting as described in our previous study (25). The membranes were immersed in a 5% non-fat milk solution (cat. no. A0830,0500; PanReac AppliChem) in TBS buffer with pH 7.5, consisting of Tris (20 mM) and NaCl (500 mM), supplemented with Tween-20 (0.1%) at room temperature over a period of 30 min to prevent non-specific absorption. Subsequently, the membranes were incubated with primary antibodies overnight at 4˚C. The primary antibodies targeting phosphorylated (p)-AKT (cat. no. 9271), AKT (cat. no. 9272), p-Ribosomal Protein S6 Kinase B1(S6K) (cat. no. 9205), S6K (cat. no. 2708) and ERα (cat. no. 8644) (all diluted at 1:1,000; all from Cell Signaling Technology, Inc.) were employed, with antibodies against α-tubulin (1:1,000; cat. no. 2144; Cell Signaling Technology) serving as loading controls. Appropriate IgGs (1:10,000; cat. no. 111-035-003; Jackson ImmunoResearch Europe) conjugated with horseradish peroxidase at room temperature during an hour were used as secondary antibodies. Signal detection was achieved using ECL reagents prepared according to Mruk's protocol (26) by ourselves, and the ImageQuant LAS4000 system for chemiluminescence (GE HealthCare) was utilized. Densitometry for the tested proteins/α-tubulin ratio was carried out using ImageJ 1.53q software (National Institutes of Health). The protocol for densitometry was provided by The University of Queensland, with recommendations from the references (27,28).

### Statistical analysis

Each antiproliferative assay was independently replicated three times, with each replication comprising three technical replicates. Statistical analysis was performed using Microsoft Excel 2019 (Microsoft Corp.) and GraphPad 9.0 software (Dotmatics). The IC_50_ value was calculated to determine the concentration of tamoxifen to produce 50% inhibition of cell growth. The results were presented as the mean ± standard deviation (S.D.), unless otherwise specified. P<0.05 was considered to indicate a statistically significant difference.

## Results

### 5-FU-induced DNA damage

**Figure 1:**
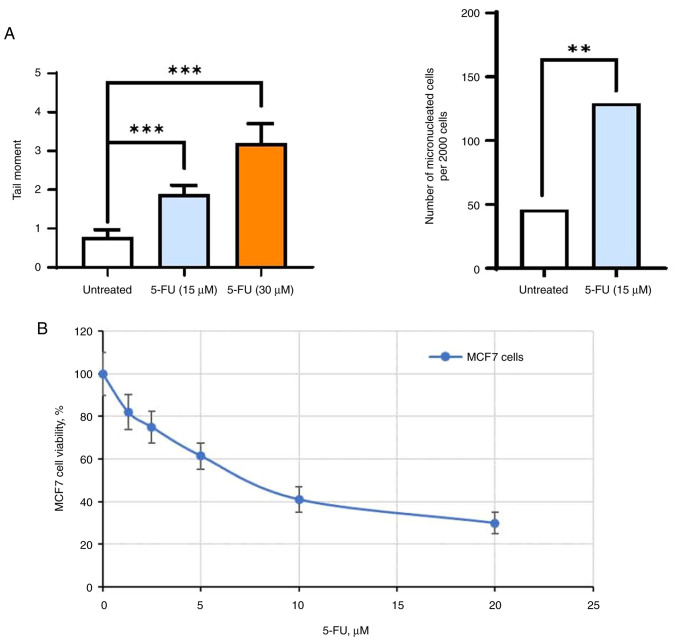
MCF7 cells response to 5-FU. (A) The MCF7 cells were treated with 5-FU at the indicated doses and the efficiency of 5-FU-induced DNA damage was evaluated using the DNA fragmentation test-Comet assay, and by the accumulation of micronuclei in cells. The significance of differences in the damage level between untreated and treated cells was calculated using Pearson's χ^2^ test. (B) The sensitivity of MCF7 cells to 5-FU treatment. MCF7 cells were treated with 1.25-20 µM 5-FU for three days and the cell viability was assessed by the MTT assay. Data represent the mean value ± SD of three independent experiments. Percentage of 100% was set as the viability of MCF7 cells treated with vehicle control. ^**^P<0.01 and ^***^P<0.0001. 5-FU, 5-fluorouracil.

The primary objective of this experiment was to investigate the impact of DNA damage agents on estrogen signaling and the subsequent sensitivity of breast tumors to hormonal therapy. Specifically, the authors focused on 5-FU (29-31), a cytostatic chemotherapeutic drug widely employed in breast cancer treatment. The experiments were conducted on *in vitro*-cultured MCF7 breast cancer cells. The effectiveness of 5-FU-induced DNA damage was assessed using the DNA fragmentation test, specifically the Comet assay, and by measuring the accumulation of micronuclei in cells as an outcome of DNA disruption. As demonstrated, a single exposure of MCF7 cells to 5-FU resulted in notable DNA fragmentation and the accumulation of micronuclei within cells (Figs. 1A and S1A and B), correlating with a substantial decrease in the number of viable cells (Fig. 1B). To elucidate whether such DNA damage can disrupt ERα signaling and to determine the duration of such alterations, an in-depth analysis of estrogen signaling and the responsiveness to hormone therapy in 5-FU-treated breast cancer cells was conducted.

### Influence of 5-FU on ERα signaling and cell response to antiestrogen tamoxifen

**Figure 2:**
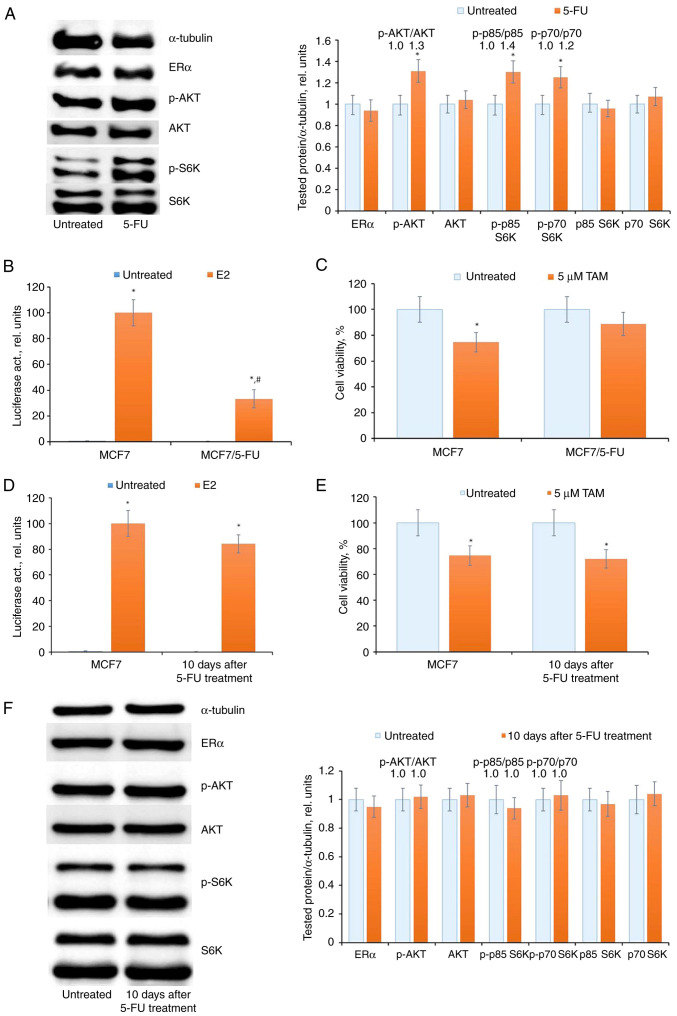
5-FU treatment and ERα signaling in MCF7 cells. (A) Western blot analysis of ERα, p-AKT, AKT, p-p85/p-p70 S6K and p85/p70 S6K in cell extracts. The MCF7 cells were treated with 15 µM 5-FU for three days and the cells were subjected to western blotting. Protein loading was controlled by membrane hybridization with α-tubulin antibodies. The blot represents the results of one of three similar experiments. Densitometry for the tested proteins/α-tubulin ratio was carried out using ImageJ software (right diagram). ^*^P<0.05. (B) Reporter analysis of ERα transcriptional activity. The cells were treated with 15 µM 5-FU for three days, then the cells were transfected with the plasmid containing the luciferase reporter gene under estrogen-responsive elements, and β-galactosidase plasmid. The cells were treated with or without 10 nM 17β-estradiol (E2) for 24 h, and the luciferase and β-galactosidase activities were determined. The relative luciferase activity was calculated in arbitrary units as the ratio of luciferase to the β-galactosidase activity. A total of 100 relative units were set as the luciferase activity in MCF7 cells treated with E2. Data represent the mean values ± S.D. of three independent experiments: ^*^P<0.05 vs. untreated samples; ^#^P<0.05 vs. E2-treated MCF7 cells. (C) Cell sensitivity to antiestrogen tamoxifen. The cells were treated with 15 µM 5-FU for three days following 5-FU withdrawal for three days. Then MCF7 cells were treated with 5 µM tamoxifen for three days and the number of viable cells was assessed by the MTT-test. Data represent the mean value ± SD of three independent experiments. Percentage of 100% was set as the viability of untreated cells. ^*^P<0.05 vs. untreated samples. (D) Analysis of luciferase activity in MCF7 cells after 5-FU withdrawal. The cells were treated with 15 µM 5-FU for three days following 5-FU withdrawal for ten days: ^*^P<0.05 vs. untreated samples. (E) The cell response to tamoxifen. ^*^P<0.05 vs. untreated samples. (F) Western blot analysis of ERα, p-AKT, AKT, p-S6K, and S6K in control MCF7 cells and MCF7 cells 10 days after treatment. 5-FU, 5-fluorouracil; ERα, estrogen receptor α; 5-FU, 5-fluorouracil; S6K, Ribosomal Protein S6 Kinase B1; p-, phosphorylated.

MCF7 cells were subjected to a three-day treatment with 5-FU, followed by the assessment of ERα expression and activity. Western blot analysis revealed non-significant changes in ERα expression in 5-FU-treated cells, while reporter analysis of ERα transcriptional activity exhibited a significant suppression following 5-FU exposure. Simultaneously, an activation of AKT, p85 S6K and p70 S6K phosphorylation in 5-FU-treated cells was observed, suggesting a potential compensatory reaction to the inhibition of ERα signaling (Fig. 2A and B). In parallel, the analysis of cell sensitivity to the antiestrogen tamoxifen indicated a decrease in cell sensitivity to the growth inhibitory effects of tamoxifen (Fig. 2C). The IC_50_ values of tamoxifen were 7.2±0.9 and 12.1±1.3 µM for MCF7 and MCF7/5-FU respectively. Upon withdrawal of 5-FU and the transfer of cells to a standard medium for ten days, there was a notable restoration of ERα transcriptional activity and cell sensitivity to the antiestrogen tamoxifen (Fig. 2D and E). This restoration was concomitant with a reduction in AKT and p85 S6K and p70 S6K phosphorylation levels (Fig. 2F).

### Effect of prolonged 5-FU treatment on the ERα machinery

**Figure 3:**
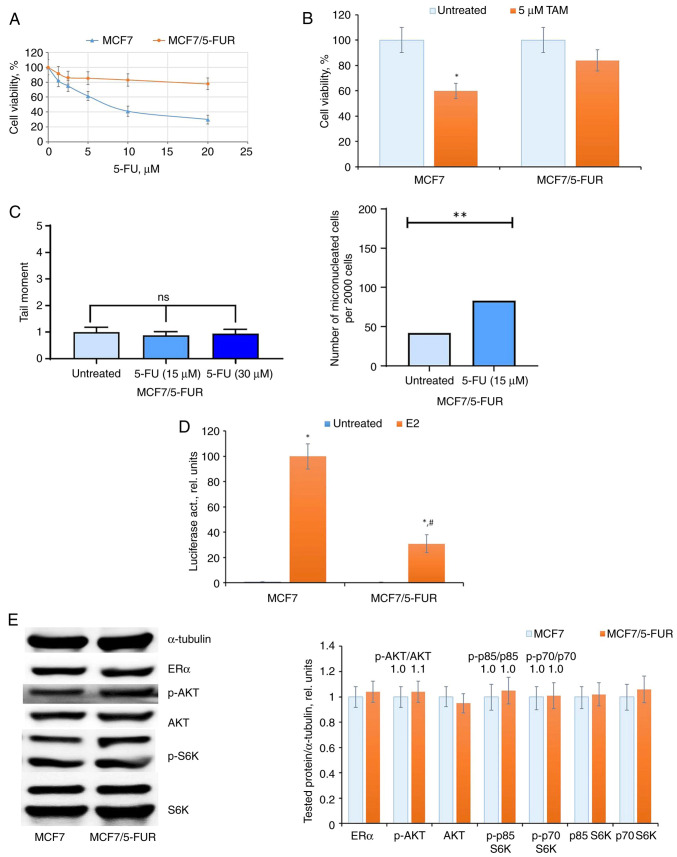
Prolonged 5-FU treatment and selection of 5-FU-resistant cells. The MCF7 cells were treated with 15 µM 5-FU within two months with subsequent 5-FU withdrawal and cell cultivation in medium without drug for the next one month. (A and B) The sensitivity of the established MCF7/5-FUR cells to (A) 5-FU, (B) tamoxifen and (C) DNA damage tests (Comet assay and accumulation of micronuclei). (D and E) ERα signaling in 5-FU-resistant MCF7/5-FUR cells. (D) Reporter analysis of ERα and (E) western blot analysis of ERα, p-AKT, AKT, p-S6K, and S6K expression in MCF7 and MCF7/5-FUR cells. ^*^P<0.05 and ^**^P<0.01 vs. untreated samples; ^#^P<0.05 vs. E2-treated MCF7 cells activity. ERα, estrogen receptor α; 5-FU, 5-fluorouracil; 5-FUR, 5-FU resistant; S6K, Ribosomal Protein S6 Kinase B1; TAM, tamoxifen; p-, phosphorylated.

To explore the impact of repeated courses of chemotherapy, the effect of prolonged 5-FU treatment was examined on ERα signaling in MCF7 cells. These cells underwent a two-month treatment with 5-FU, followed by withdrawal of 5-FU and cultivation in standard medium for an additional month. The resulting cell subline, designated as MCF7/FUR, exhibited a notable resistance to 5-FU (Fig. 3A), and significantly, demonstrated marked resistance to antiestrogen tamoxifen (Fig. 3B). The comparative analysis of 5-FU-induced DNA damage revealed a decreased response in the resistant subline to 5-FU treatment (Fig. 3C).

In the analysis of ERα machinery, a suppression of ERα transcriptional activity was evident in 5-FU-resistant cells (Fig. 3D). Subsequent examination of growth-related signaling proteins indicated no significant changes either in ERα expression or in the level of AKT and S6K signaling in the resistant cells (Fig. 3E).

### UVC irradiation and ERα signaling

**Figure 4:**
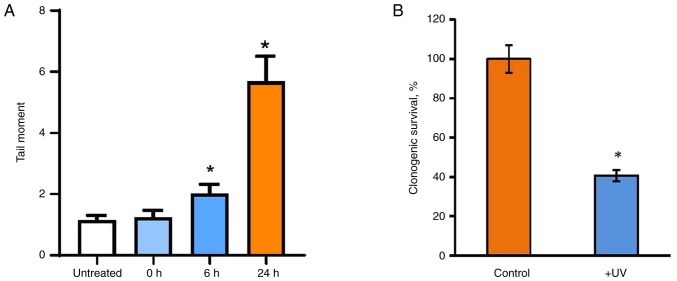
UV influence on the viability of MCF7 cells. (A and B) The cells were exposed to a single UVC dose, and after 0-24 h (A) Comet assay (the significance of differences in the damage level between control and UV-exposed cells was calculated using Pearson's χ^2^ test; ^*^P<0.05 vs. untreated and ‘0 h’ samples) and (B) colony-forming test (^*^P<0.05 vs. control samples) (b) were performed. UV, ultraviolet.

The question of whether the 5-FU-induced suppression of estrogen signaling is a shared event following DNA damage or if these alterations are unique to 5-FU was addressed in the subsequent experiments. The impact of UVC irradiation as a commonly used DNA damage agent was examined on the estrogen signaling of MCF7 cells. The results revealed that UVC irradiation leads to pronounced DNA fragmentation and a reduction in the number of viable cells (Figs. 4A and B and S2A and B), albeit to a different extent compared with the effects observed after 5-FU treatment.

**Figure 5:**
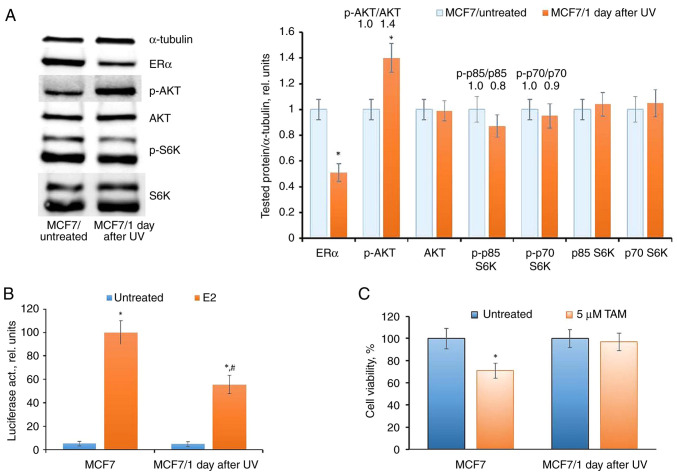
UVC influence on ERα signaling in MCF7 cells. (A-C) The cells were exposed to a single UVC dose; (A) western blot analysis of ERα, p-AKT, AKT, p-S6K and S6K (1 day after treatment, ^*^P<0.05 vs. MCF7/untreated), (B) reporter analysis of ERα (^*^P<0.05 vs. untreated samples; ^#^P<0.05 vs. E2-treated MCF7 cells) and (C) cell response to tamoxifen (3 days treatment with tamoxifen) were performed. UV, ultraviolet; ERα, estrogen receptor α; S6K, Ribosomal Protein S6 Kinase B; E2, 17β-estradiol; TAM, tamoxifen; p-, phosphorylated.

The examination of ERα expression and transcriptional activity in UV-exposed cells revealed a reduction, coupled with the activation of AKT phosphorylation (Fig. 5A and B), no significant changes in the level of S6K phosphorylation were detected. Additionally, a concurrent analysis of the cell response to the growth-inhibitory action of tamoxifen revealed decreased tamoxifen sensitivity in UV-exposed cells, substantiating the suppression of ERα signaling in these cells (Fig. 5C).

**Figure 6:**
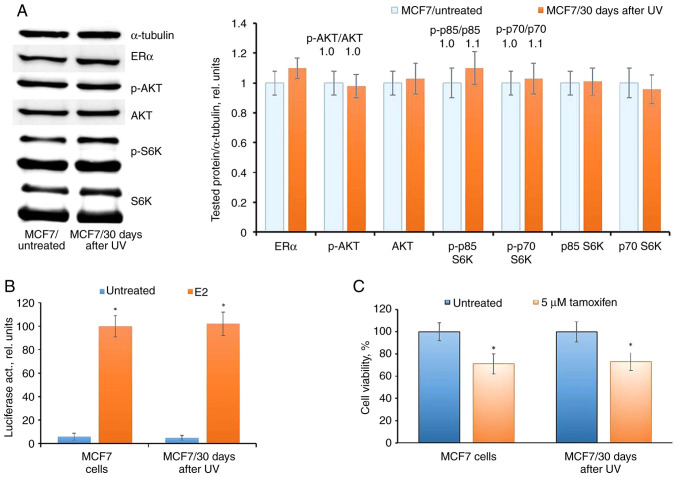
Analysis of ERα signaling in MCF7 cells 30 days after single UVС irradiation. (A) Western blot analysis, (B) reporter analysis of ERα and (C) cell response to tamoxifen were performed; ^*^P<0.05 vs. untreated samples. ERα, estrogen receptor α; UV, ultraviolet; S6K, Ribosomal Protein S6 Kinase B1; E2, 17β-estradiol; p-, phosphorylated.

The subsequent analysis conducted 30 days after UV irradiation demonstrated a complete restoration of ERα expression and activity, alongside an unchanged level of AKT phosphorylation. This restoration was correlated with the regained sensitivity of cells to tamoxifen (Fig. 6A-C).

### Selection and characterization of UV-resistant clones

**Figure 7:**
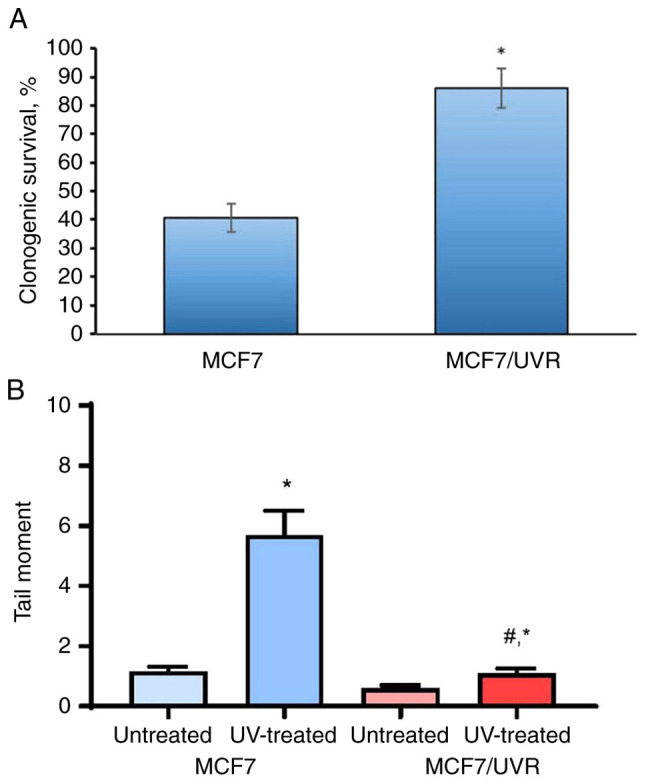
Selection and cell viability of UV-resistant subline. The MCF7 cells were exposed to UVC once every three days for 4 weeks with subsequent cell growth in standard medium for the next 40 days. (A and B) The comparative analysis of the cell viability of the parent MCF7 cells and the established MCF7/UVR subline was performed using (A) colony-forming test (^*^P<0.05 vs. MCF7 cells) and (B) Comet assay (the difference between control and UV-exposed cells was calculated using Pearson's χ^2^ test) (^*^P<0.05 vs. untreated cells; ^#^P<0.05 vs. UV-treated MCF7 cells). UV, ultraviolet.

To explore the impact of continuous UV irradiation on estrogen signaling, MCF7 cells underwent repeated UV exposure once every three days for 4 weeks, followed by the maintenance of cell growth for at least 40 days after the last irradiation. The analysis of UV sensitivity in the selected cells, denoted as MCF7/UVR, revealed a significant increase of cell survival under UV compared with the UV-treated parent MCF7 cells (Fig. 7A). UVC irradiation of MCF7 induced pronounced DNA fragmentation, while no significant difference in DNA damage was observed in MCF7/UVR compared with the untreated control (Fig. 7B).

**Figure 8:**
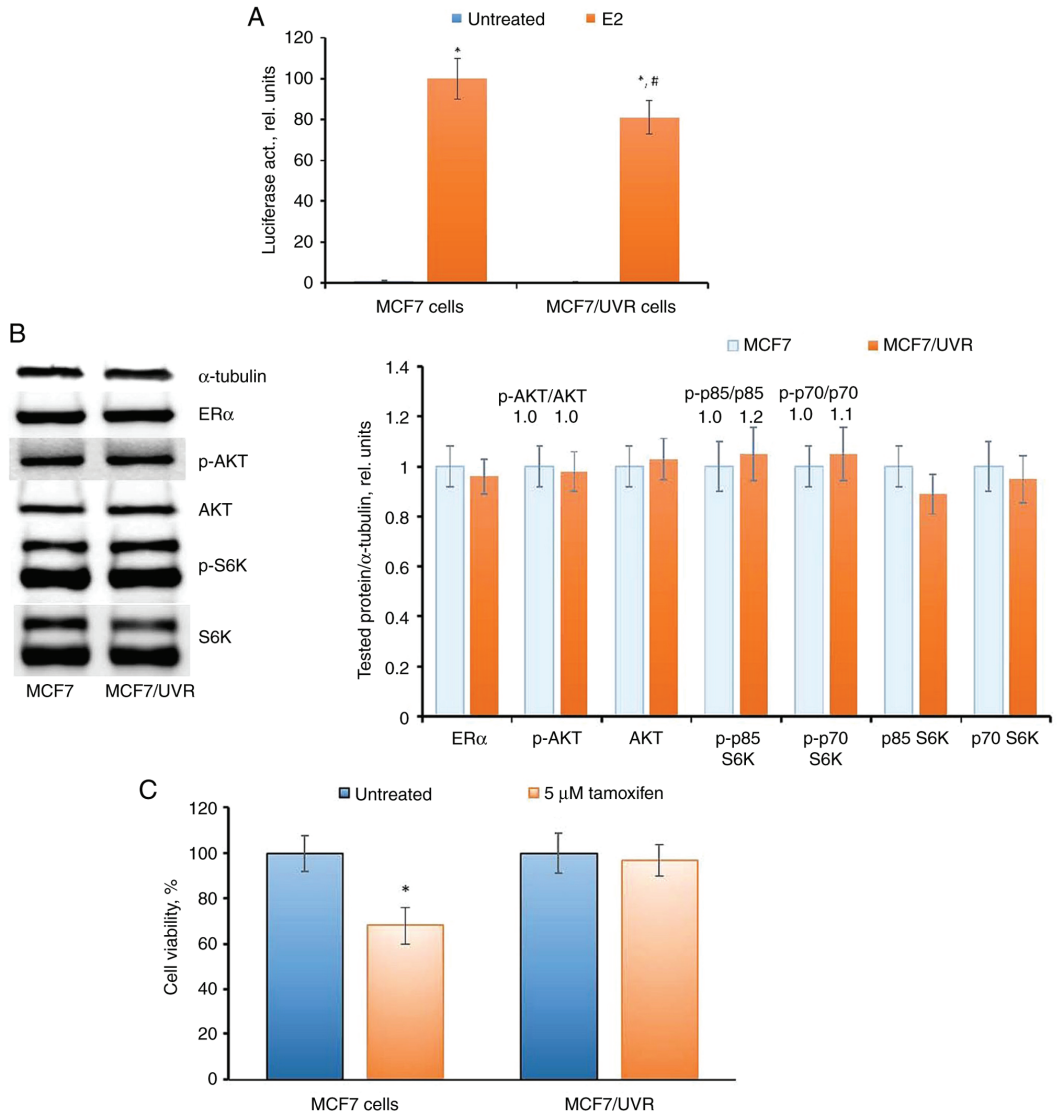
ERα signaling in MCF7/UVR cells. (A) Reporter analysis of ERα (^*^P<0.05 vs. untreated samples; ^#^P<0.05 vs. E2-treated MCF7 cells), (B) western blot analysis and (C) cell sensitivity to tamoxifen (^*^P<0.05 vs. untreated samples). ERα, estrogen receptor α; UV, ultraviolet; p-, phosphorylated.

MCF7/UVR cells exhibited an irreversible reduction in ERα transcriptional activity (Fig. 8A), despite the restored level of ERα expression (Fig. 8B). Examination of the AKT pathway did not reveal changes in the corresponding signaling proteins. The analysis of the cell response to tamoxifen indicated that MCF7/UVR cells retained partial resistance to tamoxifen for at least 40 days after irradiation (Fig. 8C), in contrast to the parent MCF7 cells after a single UV dose.

## Discussion

Hormone therapy (32-36) is extensively employed in the treatment of hormone-dependent breast tumors, either as a monotherapy or more frequently in combination with chemotherapy or radiotherapy. While the action of most chemotherapy drugs is linked to DNA damage, the exact role of DNA damage in influencing the estrogen receptor machinery in tumor cells remains unclear.

Several studies have highlighted alterations in the estrogen receptor status of breast tumors following neoadjuvant chemotherapy (13). Additionally, there has been evidence of the overexpression of miRNAs targeting the estrogen receptor after neoadjuvant chemotherapy (15). Furthermore, a correlation has been described between the decreased expression of DNA repair genes and the emergence of hormone resistance in breast cancer cells (37).

UV irradiation (38-41) serves as a widely utilized experimental model for investigating cellular responses to DNA damage treatment. Evidence has been accumulated regarding the influence of UV irradiation on the activity of various cell signaling proteins, including but not limited to p38 MAPK, Jun N-terminal kinase, extracellular signal-regulated kinase 1/2, NF-κB (42,43), eIF2α (44), Toll-like receptors (45), HER2/neu (46), death domain-associated protein (DAXX) (47), and others. In further studies, potential p42/44 ERKs-, AKT- and p38-mediated phosphorylation of ERα in UVC-treated cells is to be investigated. UV irradiation has been indicated to stimulate the bystander effect (48), with corresponding events such as apoptosis, premature senescence, single and double DNA strand breaks, and reduced clonogenic survival described in bystander cells (49).

However, contradictory findings exist, regarding the relationship between DNA damage and hormonal resistance. Various data suggest that radiation-induced DNA damage either does not lead to or is associated with only a marginal increase in overall survival for patients with ERα-negative breast cancer (50,51). In patients with ERα-positive breast cancer, no significant trend in this regard has been consistently identified (52-54). In studies involving *in vitro*-cultured breast cancer cells, previous studies have revealed a correlation between radiation exposure and disruptions in hormonal cell signaling. These disruptions include a partial loss of ERα and the development of resistance to antiestrogen (16,17). Furthermore, a relationship has been identified between the development of acquired radioresistance and hormonal resistance in breast cancer cells, providing general support for the possibility of impairment in hormonal signaling during irradiation (55-58).

The primary objective of the present study was to explore the impact of DNA damage agents on estrogen signaling and the sensitivity of breast cancer cells to hormonal drugs. The findings of the present study indicated that the response of MCF7 breast cancer cells to 5-FU was linked to alterations in estrogen signaling and the activation of the bypass AKT signaling pathway. A single treatment with 5-FU induces temporary changes in AKT signaling pathways, whereas chronic 5-FU exposure leads to the selection of 5-FU-resistant cells exhibiting irreversible alterations in ERα signaling, correlated with partial hormonal resistance. Similar alterations were observed in cells subjected to UV-induced DNA damage, emphasizing the pivotal role of DNA damage in modifying ERα signaling in breast cancer cells. These observed changes persist in cells for several months after drug treatment, suggesting the potential involvement of (epi)genetic machinery in maintaining the resistant phenotype. ERα and AKT kinase are among the key regulators of breast cancer cell proliferation. Significant efforts of researchers are directed towards the development of novel inhibitors of these targets. The development of such inhibitors also takes into account the significant overlap between signaling pathways. The signaling between ERα and AKT axis largely determines the formation of resistance to targeted and hormonal therapies, and the assessment of these parameters is important for disease prognosis and, in some cases, for changing treatment protocols (59-61). Interestingly, AKT overexpression leads to upregulation of estrogen-regulated pS2 gene, Bcl-2, and macrophage inhibitory cytokine 1(62). Moreover, AKT protects breast cancer cells from tamoxifen-induced apoptosis. The AKT-mediated activation of ERα in 5-FU treated cells has not been described in detail in the present study, and is of great interest for further study, including by means of CRISPR/Cas9 technology.

Additional investigations are required to elucidate the mechanism by which DNA damage agents deactivate estrogen receptors. The present findings suggested that the inhibition of ERα transcriptional activity induced by drugs/UV is not correlated with corresponding changes in ERα expression. This underscores the crucial role of post-translational modifications in the regulation of ERα. The reduction in ERα transcriptional activity may stem from an imbalance between ERα co-activators and corepressors induced by DNA damage agents. Evidence supporting this includes the observed suppression of ERα co-activator CBP/p300 in response to 5-FU (63) and the modulation of ERα coregulator MDC1 (mediator of DNA damage checkpoint 1) in response to DNA damage (64). Similarly, several studies have highlighted the involvement of ERα coregulators (65) and ERα-binding chaperones (66) in the cellular response to irradiation-induced DNA damage agents. Significantly, MCF7 cells are characterized as p53-positive tumor cells, suggesting potential interactions between p53 and ERα signaling. Currently, only few studies describe the interrelation between p53 and ERα, highlighting changes in p53 activity under estrogen stimulation (67,68). MCF7 cells are wtERα and wtp53 positive (69) but the interplay between the two transcription factors in 5-FU and UVC-treated cells has not been investigated in the present study. Additionally, a series of observations underscore the involvement of growth-related pathways, including PI3K/AKT and MAP cascades, in the regulation of ERα activity. Moreover, the role of ERα itself has been implicated in the regulation of cellular radioresistance (58,70,71). Further studies are required for the explanation of the mechanism of the inactivation of ERα by DNA damage agents and how DNA damage inhibits ERα transcription without affecting its expression. In addition, an extension of the study is possible with the use of a tamoxifen gradient, as it is known that the effects of tamoxifen vary greatly depending on the dose used.

In conclusion, the present findings suggested that the treatment of cancer cells with DNA damage agents may lead to the irreversible suppression of estrogen signaling and the progression of partial hormonal resistance, thus limiting the efficiency of combined or subsequent chemo- and hormone therapy.

## Supplementary Material

Effects of 5-FU on MCF7 cells. (A) Light microscopy images demonstrating morphology of MCF7 cells after 5-FU treatment [Inverted microscope Diavert (Leitz), phase-contrast objective Phaco x20, camera DP-70 with software DP-controller (Olympus Corporation)]. (B) Comet assay indicating DNA damage in 5-FU-treated MCF7 cells. The slides were observed with a Zeiss AxioVert 200 (Carl Zeiss AG) fluorescence microscope with an EBQ isolated lamp at x10 magnification. 5-FU, 5-fluorouracil.

Effects of UVC on MCF7 cells. (A) Light microscopy images demonstrating morphology of MCF7 cells after UVC exposure. (B) Comet assay indicating DNA damage in MCF7 cells after UVC exposure. The slides were observed with a Zeiss AxioVert 200 fluorescence microscope with an EBQ isolated lamp at x10 magnification. UV, ultraviolet.

## Data Availability

The datasets used and/or analyzed during the current study are available from the corresponding author upon reasonable request.
