# Supplementary material for: Irreversible inhibition of estrogen receptor α signaling and the emergence of hormonal resistance in MCF7 breast cancer cells induced by DNA damage agents
Source: Biomed Rep. 2024 Jan 19;20(3):42. doi: 10.3892/br.2024.1727 (PMC10853760; doi:10.3892/br.2024.1727)

Figure S1. Effects of 5-FU on MCF7 cells. (A) Light microscopy images demonstrating morphology of MCF7 cells after 5-FU treatment [Inverted microscope Diavert (Leitz), phase-contrast objective Phaco x20, camera DP-70 with software DP-controller (Olympus Corporation)]. (B) Comet assay indicating DNA damage in 5-FU-treated MCF7 cells. The slides were observed with a Zeiss AxioVert 200 (Carl Zeiss AG) fluorescence microscope with an EBQ isolated lamp at x10 magnification. 5-FU, 5-fluorouracil.

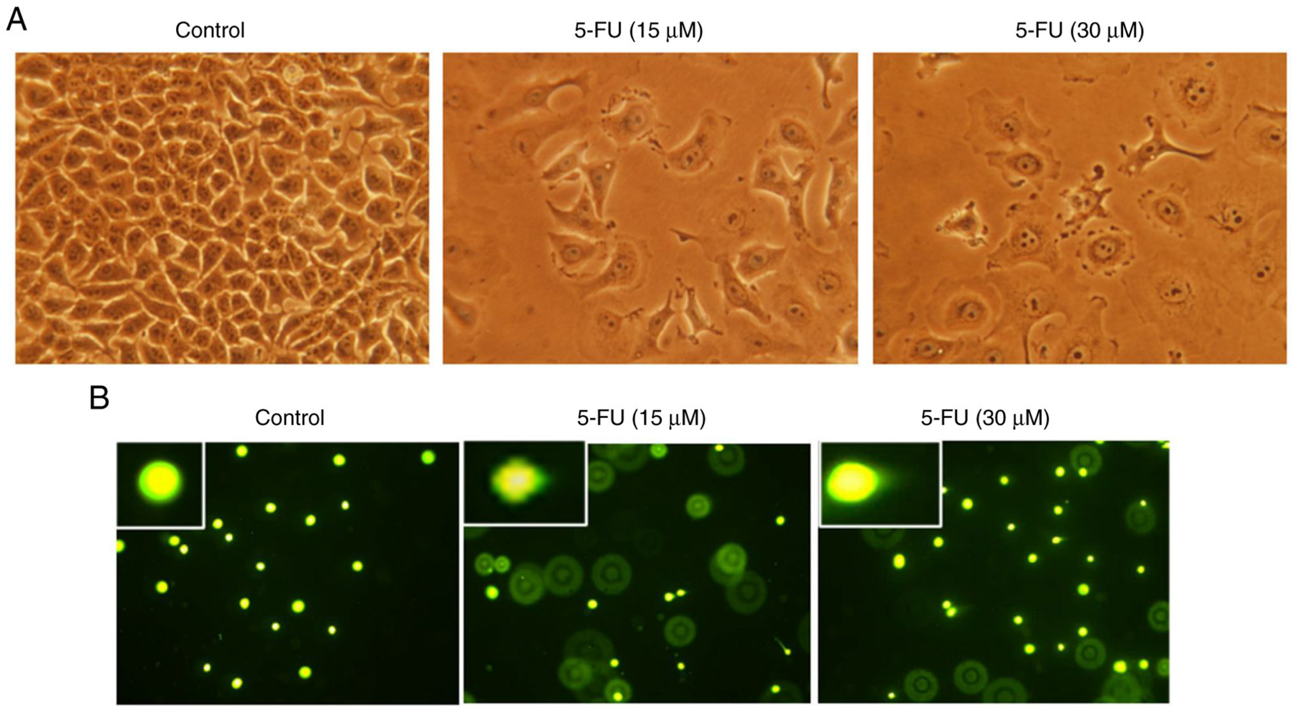

Figure S2. Effects of UVC on MCF7 cells. (A) Light microscopy images demonstrating morphology of MCF7 cells after UVC exposure. (B) Comet assay indicating DNA damage in MCF7 cells after UVC exposure. The slides were observed with a Zeiss AxioVert 200 fluorescence microscope with an EBQ isolated lamp at x10 magnification. UV, ultraviolet.

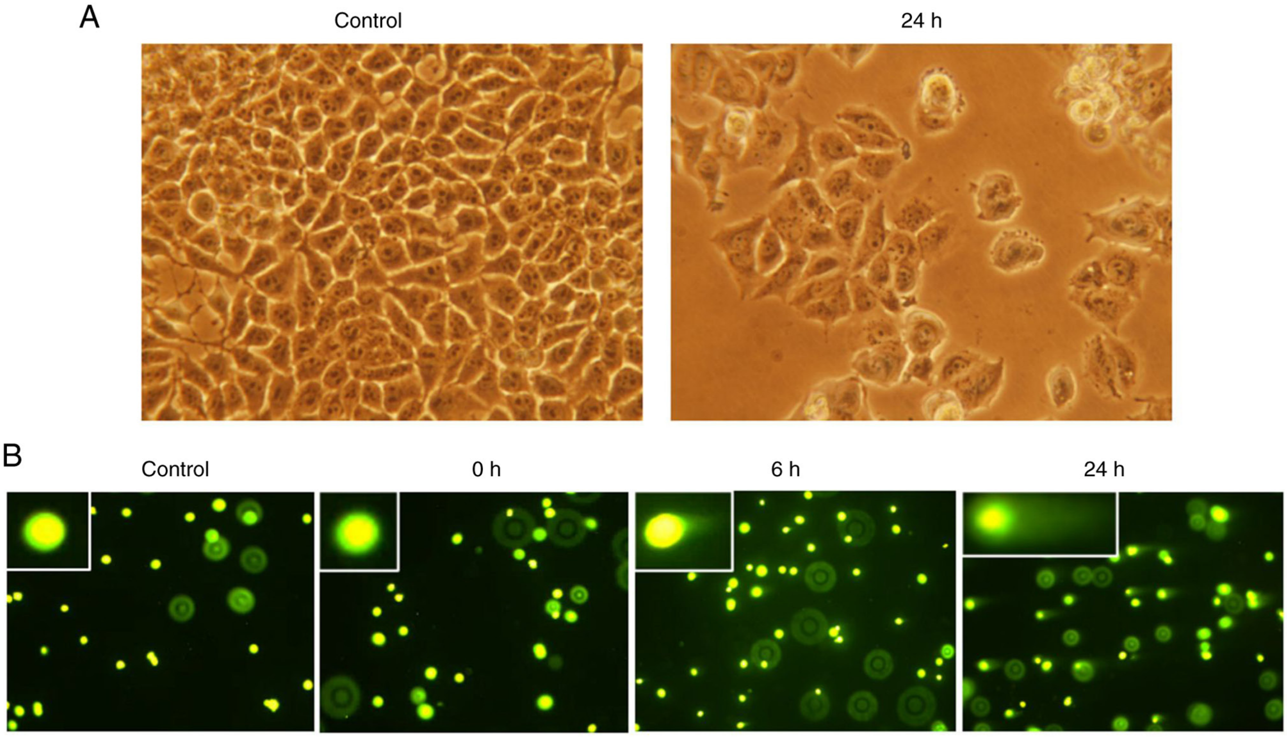

Supplement: Effects of 5-FU on MCF7 cells. (A) Light microscopy images demonstrating morphology of MCF7 cells after 5-FU treatment [Inverted microscope Diavert (Leitz), phase-contrast objective Phaco x20, camera DP-70 with software DP-controller (Olympus Corporation)]. (B) Comet assay indicating DNA damage in 5 [file Supplementary_Data.pdf]
